# Application of 3D Printing in Training Health Care Providers; the Development of Diverse Facial Overlays for Simulation-Based Medical Training

**DOI:** 10.7759/cureus.26637

**Published:** 2022-07-07

**Authors:** Julia Micallef, Anusha Broekhuyse, Sanjana Vuyyuru, Randy Wax, S K Sridhar, Jane Heath, Suhair Clarke, Adam Dubrowski

**Affiliations:** 1 Faculty of Health Sciences, Ontario Tech University, Oshawa, CAN; 2 Faculty of Engineering, McMaster University, Hamilton, CAN; 3 Faculty of Science, Western University, London, CAN; 4 Critical Care Medicine, Lakeridge Health, Oshawa, CAN; 5 Respiratory Medicine, Lakeridge Health, Oshawa, CAN; 6 Medical Education and Simulation, Lakeridge Health, Oshawa, CAN

**Keywords:** task-trainer, simulation based training, simulation-based-education, simulation in medical education, three-dimensional (3d) printing, diversity and inclusion, additive manufacturing

## Abstract

The medical simulation manikins used by healthcare learners provide the training of numerous clinical skills but often lack diversity with respect to race, ethnicity, age, and sex. Having a diverse medical education environment is imperative for exposing learners to the diverse population of patients they may encounter when in practice. In this technical report, the development of diverse and cost-effective facial overlays produced using 3D scanning, 3D printing, and silicone to be used on top of the current medical manikins at Lakeridge Health Hospital (Oshawa, Ontario, Canada) is described. To obtain consistent feedback throughout the development process, an advisory committee was consulted monthly at Lakeridge Health Hospital. The process began by determining that two facial overlays would be developed based on the two groups that represent the highest percentage of visible minorities in the Durham Region (Ontario, Canada). Facial overlays representing the South Asian (31.8%) and Black (29.6%) races were chosen. To prevent the generalizability of the facial features of these two races, volunteers who identified as specific ethnicities (East Indian and Jamaican) within each race were selected. To add variation in age for the facial overlays, the East Indian facial overlay was edited to represent an adolescent teenager (15 to 17 years old) and the Jamaican overlay was edited to represent an elderly citizen (over 60 years old). The facial overlays were developed from the 3D scans of the two volunteers and were used to create the design of 3D printed molds, in which silicone was poured in. Pigments were added to the silicone to match the skin tones of the two volunteers, and these specific tones were used as the base color for each facial overlay. Details, such as wrinkles, eyebrows, and lip color, were painted on top of the base using additional pigmented silicone. Additionally, neck overlays were created to provide continuity of the skin tone of the facial overlay. To retain the functionality of the medical manikins, the eyes of the facial overlays were cut out, and the mouth was cut open to allow for intubation training. For stability purposes, Velcro attachments were added to the facial and neck overlays so that they could be secured onto the medical manikins. Overall, the costs to manufacture both facial overlays resulted in CAD 235.65, including local taxes. Once manufactured, both facial overlays were tested by medical students (n=18) during two separate advanced cardiovascular life support (ACLS) training sessions in the local, hospital-based simulation laboratory at Lakeridge Health Hospital. The feedback obtained suggested a need to improve the functionality of the facial overlays by making the mouths bigger and less stiff for easier intubation. However, the overlays were accepted overall as a means to add diversity to the current medical manikins. In the end, cost-effective and diverse facial overlays were created to be used on top of the medical manikins that are currently being used by healthcare learners at Lakeridge Health Hospital.

## Introduction

Simulation-based medical education (SBME) is a rapidly growing field that employs realistic simulators to allow healthcare professionals, educators, and learners to practice clinical procedures in a safe and controlled environment without causing unnecessary patient harm [1]. For example, healthcare learners of various specialties use task training medical manikins to practice procedures such as intubation. While these medical manikins provide the opportunity for medical students to practice numerous skills deemed necessary for them to enter the clinical setting, they often lack racial, ethnic, sex, and age diversity [2,3]. This gap in SBME is concerning as exposure to various races, ethnicities, sexes, and ages are important aspects of health care education, and there is a variation of conditions, treatments, and diagnoses associated with these different population groups that the learners are not being exposed to [2,3]. 

Currently, most medical manikins are healthy, adult Caucasian males [3] and thus, do not represent the patient population in the Durham Region (Ontario, Canada). Although diverse simulators are starting to emerge in the market, they are still limited in terms of available races and ethnicities to choose from. These simulators just have different skin tones with the same physique of a healthy, adult Caucasian male, thus emphasizing the lack of diverse simulators on the market in terms of race, ethnicity, sex, and age. High-fidelity medical manikins such as the SimMan®, from the company Laerdal, Norway (https://laerdal.com/ca/products/simulation-training/emergency-care-trauma/simman/), are extremely expensive, ranging in price between USD 65,000 to USD 85,000 [4]. Additionally, SimMan Facial Overlays from MedicFX, New Zealand (https://www.medicfx.com/), which meet these diverse needs described above, cost approximately CAD 1,200 without taxes [5].

To address the lack of diversity and the high cost of medical manikins, an alternative approach is proposed in this technical report - SBME with diverse and cost-effective facial overlays, produced using additive manufacturing (AM) technologies such as 3D scanning, 3D printing, and silicone. The AM technologies used to develop the facial overlays have three benefits. First, it allows healthcare learners to improve their clinical skills in diverse scenarios that are more representative of the patient population. Secondly, it provides a parsimonious solution that will allow program directors and stakeholders to meet diversity requirements within budgetary constraints. For instance, instead of purchasing a new medical manikin with a different skin tone, it is more cost-effective to place an overlay over the current medical manikin’s face to create an immersive training experience. Finally, this approach allows the overlays to be specifically crafted so that they are catered to the patient population in any particular geographic region.

The aims of this development and quality improvement work were to a) develop two cost-effective facial overlays using AM techniques and b) use the Michigan Standard Simulation Experience Scale (MiSSES) [6] to gain feedback from endpoint users during advanced cardiovascular life support (ACLS) training course.

## Technical report

Context

The silicone facial overlays were specifically developed to be placed on top of the task training medical manikins used in the Lakeridge Health Education and Research Network (LHEARN) Centre at Lakeridge Health Hospital (Oshawa, Ontario). Lakeridge Health Hospital uses the SimMan® medical manikins, which are all healthy, adult Caucasian males, during a variety of training sessions. Health stakeholders are committed to implementing race-based data collection and reporting methods, as race-based data is not included in many Canadian information systems to date [7]. 

To obtain consistent feedback throughout the development process, an established advisory committee was consulted monthly at Lakeridge Health Hospital. This committee consisted of a multidisciplinary team of health professionals to ensure a diverse range of feedback and advice was collected for the facial overlays. Specifically, at Lakeridge Health Hospital, there were two critical care physicians (RW and KS), one respiratory therapist (JH), and one medical simulation specialist (SC) who guided throughout the development process. The diverse committee ensured that the overlay would be functional in a variety of simulation situations. The Inclusion, Diversity & Equity (IDE) group at Lakeridge Health Hospital was also consulted to obtain additional feedback.

Inputs

The following software was used to design the facial overlays: Artec Space Spider 3D scanner (Artec3D, Santa Clara, CA), Fusion360™ (Autodesk Inc., San Rafael, CA), and Ultimaker Cura 3D printing software (Ultimaker B.V., Utrecht, Netherlands). The following materials were used to construct the facial overlays: Ecotough™ polylactic acid (PLA) filament material (Mississauga, Ontario), Dragon Skin™ 10 NV silicone (Smooth-On, Macungie, PA), THI-VEX™ (Smooth-On, Macungie, PA), Ease Release 200 (Sculpture Supply Canada, Toronto, Ontario), Silc-Pig™ coloring (Smooth-On, Macungie, PA), and TKBD-01 Mini Baby Doll Silicone Paint Trial Kit (Sculpture Supply Canada, Toronto, Ontario).

Process 

**Figure 1:**
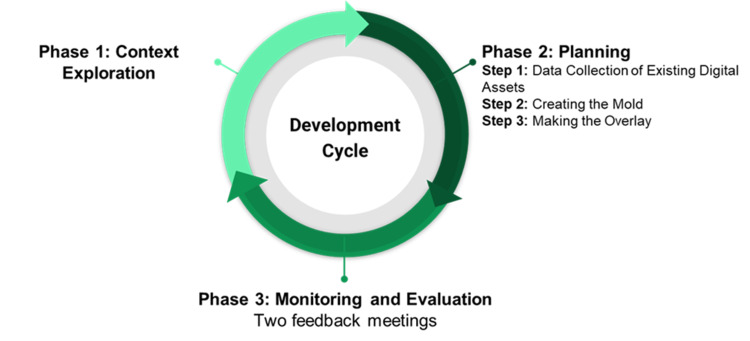
The development cycle consists of three phases to produce the facial overlays.

The Adapted Implementation Model for Simulation (AIM-SIM) was used to guide the development and feedback process [8]. This process included three distinct phases where feedback was required from the stakeholders: a) engagement and context exploration, b) planning, and c) monitoring and ongoing evaluation. The overall process is illustrated in Figure 1.

Phase 1: Engagement and Context Exploration 

During the first advisory meeting, the races and ethnicities for the overlays were selected. The demographics of the Durham Region were assessed. The Durham Region encompasses eight municipalities located east of Toronto, Ontario, and includes land that is rural, residential, and commercial [9]. It was noted that the region is becoming more ethnically diverse as 22.75% of its overall population in 2021 were visible minorities, compared to only 16.8% in 2006 [10,11]. Regions in the cities of Pickering, Ajax, and Whitby account for the highest rate of diversity, considering over 61% of students are visible minorities [10,11]. In Ajax, Pickering, and Whitby, 48.7%, 39.1%, and 22.2% of students in these cities, respectively, belong to a visible minority [10,11]. Overall, in Durham Region, 31.8% of students are South Asian, 29.6% are Black, 8.4% are Filipino, 7.0% are Chinese, 3.8% are West Asian, and 3.5% are Latin American, as summarized in Table 1 [10,11].

**Table 1:** A summary of the demographics of the visible minorities in Durham Region.

	Ethnicity	Demographics
1	South Asian	31.8% of visible minorities in Durham Region
2	Black	29.6% of visible minorities in Durham Region
3	Filipino	8.4% of visible minorities in Durham Region
4	Chinese	7.0% of visible minorities in Durham Region
5	Latin American	3.5% of visible minorities in Durham Region
6	West Asian	3.8% of visible minorities in Durham Region

Based on these demographics, two facial overlays representing the South Asian population and Black population were chosen to be developed, as these two races accounted for the highest proportion of students belonging to a visible minority in the Durham Region. The proportion of students that are South Asian is 31.8%, and the proportion of students that are Black is 29.6% [10,11]. The age and gender of the facial overlays were determined based on the availability of digital assets, 3D scanning volunteers, and technical ability of production. For the final facial overlays, an East Indian female was selected to represent an ethnicity within the South Asian race, and a Jamaican male was selected to represent an ethnicity within the Black race. The East Indian facial overlay was edited to represent an adolescent teenager (15 to 17 years old), and the Jamaican overlay was edited to represent an elderly citizen (over 60 years old).

Phase 2: Planning

Next, based on recommendations from the advisory committee and IDE group, a three-step development method was conducted:

Step 1 is data collection of existing digital assets: A preliminary search was conducted through various online 3D modeling databases to determine if realistic, diverse 3D models of human faces were available to print and aid in the creation of the overlays. Most of the online databases had very few realistic models of human faces, and any 3D models that were available were Caucasian males. The database, Turbosquid (https://www.turbosquid.com/), had a few Black 3D facial models, but there was limited selection as the models were all young males. There were no Latin American, South Asian, West Asian, East Indian, or Filipino models available for use in the database. The database, CGTrader (https://www.cgtrader.com/), had one model of a young Chinese female. Overall, this database had minimal ethnic and racial diversity in the available models. In conclusion, these 3D models were not utilized in the creation of the facial overlays as limited information was available regarding how the models were constructed, thus highlighting concern about replicability. Other databases searched include Thingiverse (https://www.thingiverse.com/), SketchFab (https://sketchfab.com/), Cults3D (https://cults3d.com/en), Pinshape (https://all3dp.com/topic/pinshape/), GrabCAD (https://grabcad.com/), 3D Export (https://3dexport.com/), and Yeggi (https://www.yeggi.com/). 

**Figure 2:**
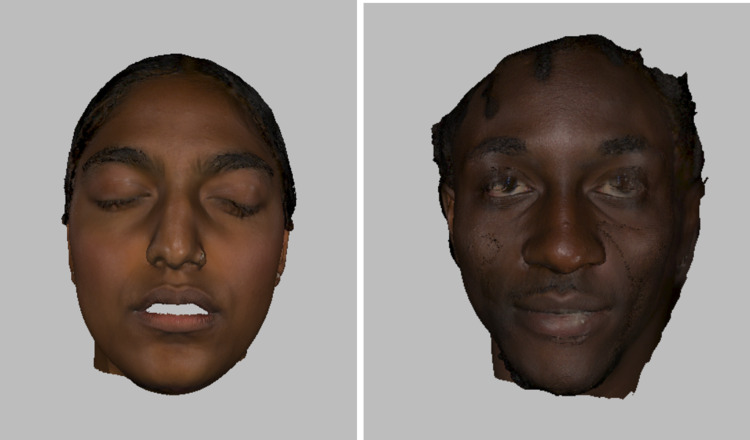
3D scan of Indian participant on the left, and the Jamaican participant on the right.

**Figure 3:**
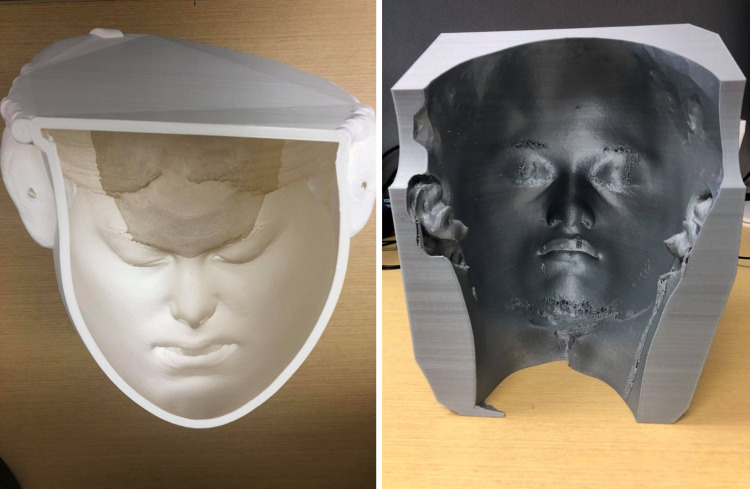
3D printed mold of Indian scan on left, and of Jamaican scan on right.

Step 2 is creating the mold: Using Fusion360™ to construct realistic faces, from scratch, with distinct facial features would be unrealistic to accomplish. As a result, it was determined that 3D scanning the faces of volunteers that identified as either South Asian or Black would be a better approach. The volunteers that identified with the races of interest were individuals that the design team knew beforehand. The volunteers were asked to sign a photo release form and gave consent to have their scans used as the base for the facial overlay designs. The Artec Space Spider 3D scanner (Luxembourg) was used to scan the faces of the volunteers and the medical manikins that medical students and learners train with. During the scanning process, volunteers were asked to have their mouths open so that the facial overlay would fit securely on the medical manikin (which had an open mouth), as shown in Figure 2. The medical manikins that were scanned were used as a sizing reference to design the facial overlay mold. The scans of the volunteers and the medical manikins were uploaded to Fusion360™, and the scan of the volunteer was scaled to be 1.5 times bigger than the size of the medical manikin. Afterward, the scan of the facial overlay was converted into a printable format. The 3D file was uploaded to the Ultimaker Cura 3D printing software, and the scan of the facial overlay was converted into a mold that the silicone was poured into. Furthermore, the 3D-rendered files were transferred to an Ultimaker S5 3D printer and were printed using Ecotough™ PLA filament material. Each mold took approximately two days to print and is shown in Figure 3.

**Figure 4:**
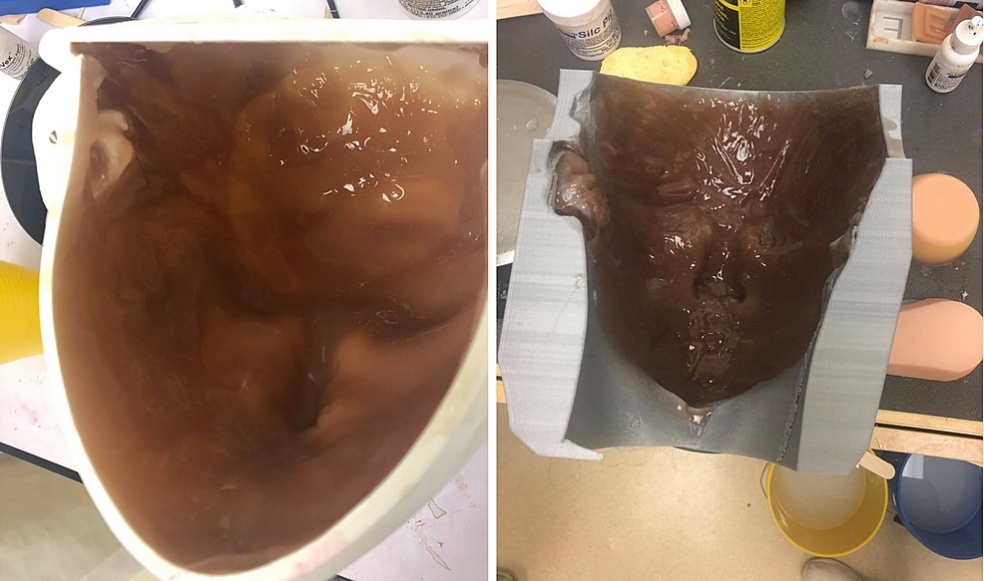
The silicone after being poured in the molds for the Indian overlay (left) and Jamaican overlay (right).

**Figure 5:**
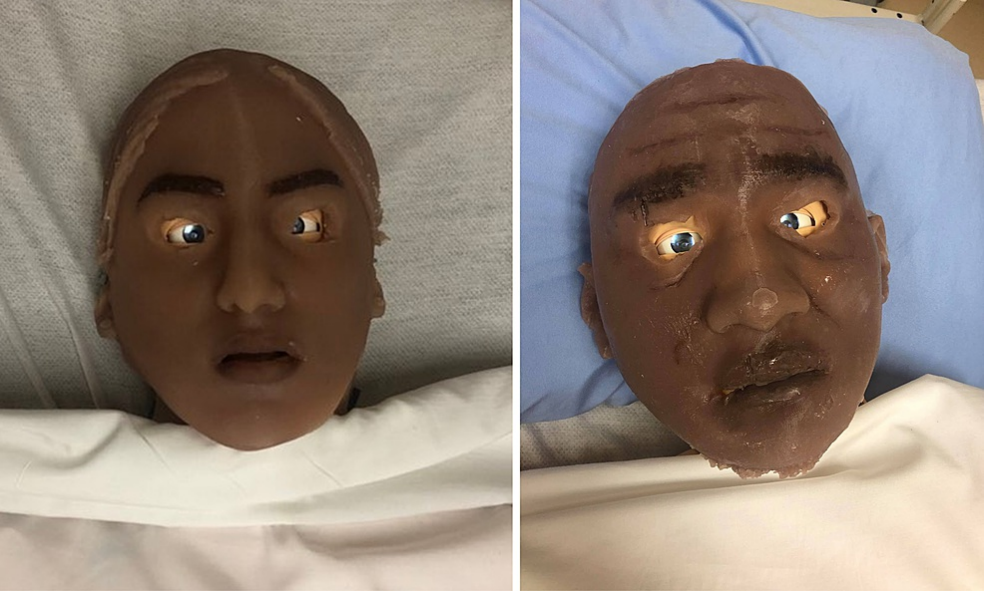
The painted Indian facial overlay on the left, and the painted Jamaican facial overlay on the right.

Step 3 is building the overlay: Once the molds for the facial overlays were printed, Ease Release™ 200 (Pennsylvania) was sprayed onto the inner surface with an emphasis on the eyes, nose, mouth, and ear areas to ensure smooth removal of the silicone. A combination of 300 g of part A and 300 g of part B of Dragon Skin™ (Pennsylvania) was used to create the silicone mix. Silc-Pig™ (Pennsylvania) silicone coloring was added by dipping one centimeter of a clean jumbo popsicle stick into the Silc-Pig™ color containers to retrieve a small amount of coloring. A single dip of Silc-Pig™ silicone coloring was added in both red and yellow to make an orange base color. This was the starting point for both the Jamaican and East Indian overlay, as it created a warm undertone for the skin color. Varying amounts of the brown Silc-Pig™ silicone color were added to the mixture until the desired skin tone was achieved. Specifically, two dips of brown coloring were added to the orange base to create the skin tone for the Jamaican overlay, and one dip of brown coloring was used to create the skin tone for the Indian overlay. Additionally, 15 drops of the silicone thickening agent, THI-VEX™ (Pennsylvania), were added to the mixture to allow the silicone to stick to the inner surfaces of the mold. All materials were mixed thoroughly for approximately three minutes. The mixture was then poured into the 3D printed molds and left to cure for approximately fifteen minutes to allow the THI-VEX™ to activate and make the mixture thicker. Once the silicone was thick enough to stick to the vertical surfaces in the inner face of the mold, the molds were tilted to get an even layer of silicone on the sides of the molds. When the silicone became too viscous to be spread through the molds via tilting, a paintbrush was used to coat the silicone onto the vertical surfaces until it stopped pooling at the bottom of the molds. This process was repeated until all surfaces of the mold had an even layer of silicone covering them, as shown in Figure 4. After the silicone was evenly applied to the entire mold, it took approximately two hours to cure. When the curing process was complete, the overlay was removed from the mold, and an opening for the mouth and eyes was cut out. Finally, to add realism to the facial overlays, details such as eyebrows, wrinkles, and lip color were added using silicone paints from the TKBD-01 Mini Baby Doll Trial Kit, as shown in Figure 5.

Phase 3: Monitoring and Evaluation

**Figure 6:**
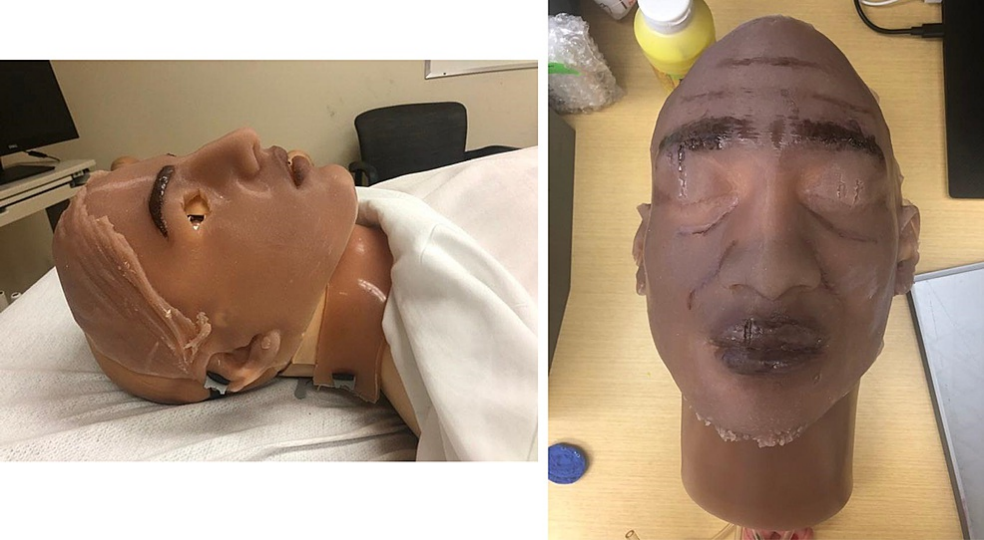
Silicone neck overlay representing Indian ethnicity shown on left, and Jamaican neck overlay shown on right.

For the second advisory meeting, silicone prototypes, and the 3D printed molds of the East Indian and Jamaican facial overlays, were presented to the advisory committee. Upon review, the committee requested that the design team print neck overlays for the medical manikin that matched the skin tone of the facial overlays, as shown in Figure 6. 

After the advisory committee’s suggestions were implemented into the overlays, a third meeting was arranged to gain a final round of feedback. The facial overlays were presented on the medical manikins to demonstrate how they would appear during simulation-based medical training sessions. A critical care physician on the committee inserted intubation equipment into the mouth of the medical manikin, with the facial overlay on top, to evaluate the durability of the overlays and assess if they would interfere with intubation training. The mouth of the Jamaican overlay was slightly small for the intubation equipment, but the mouth size of the East Indian overlay was ample and did not interfere with the equipment. Thus, the design team created a larger mouth opening for the Jamaican overlay.

**Figure 7:**
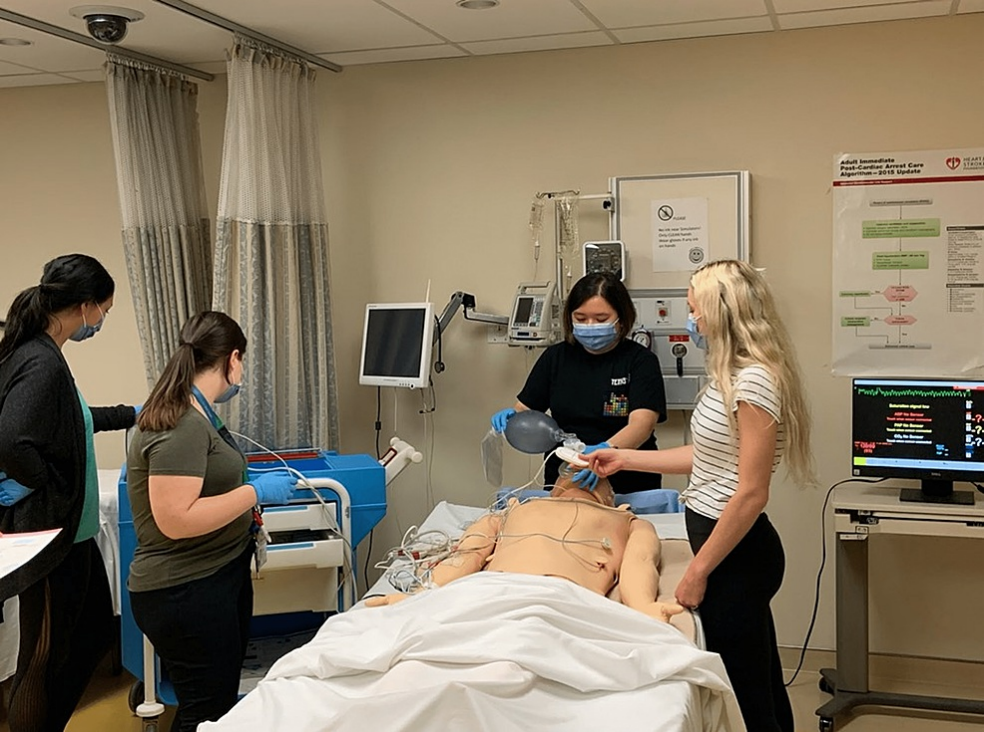
Medical students going through ACLS training using the facial overlays in the LHEARN Centre at Lakeridge Health Hospital.

Once approved by the advisory committee, both facial overlays were tested by medical students (n=18) during two separate ACLS training sessions in the LHEARN Centre at Lakeridge Health Hospital, as shown in Figure 7. In these training sessions, medical students were given a cardiovascular emergency scenario involving the simulation of medical manikins. These manikins simulated a patient that the trainees would encounter in a real-world clinical setting. The medical students treated this simulated patient by observing the information that the manikin presented, such as heart rate/EKGs, oxygen levels, and level of consciousness. Many of the scenarios involved Laryngeal mask airways and intubations, which both involve handling the mouth opening of the facial overlay and manikin. After each session, the students were given a 12-question survey (Table 2) to provide comments on the functionality and acceptability of the facial overlays. Question 5 of the survey was disregarded as this was not tested during the training session. The quantitative feedback was collected through a survey based on a modified version of the MiSSES template [6], which focused on a) perceptions related to the functionality of the facial overlays, b) the general acceptability of the facial overlays, and c) possible improvements to the facial overlays. The qualitative feedback was collected using open-ended comment boxes at the end of the survey.

**Table 2:** Survey questions given to medical students testing facial overlays after ACLS training in the LHEARN Centre at Lakeridge Health Hospital.

Question Number	Question					
1	Please rate how effectively you were able to intubate with the facial overlay.					
2	If you have difficulty with performing the intubation, please elaborate.					
3	Please rate how well you were able to see the eyes of the task trainer.					
4	Please rate how well the facial overlays stayed on top of the task trainer.					
5	Please rate how well the COVID-19 masks fit on the overlays.					
6	On a scale of 1 to 5, how realistic do the facial overlays look compared to the task trainers?					
7	Which race category best describes you? Select all that apply.					
8	In your opinion, how well do you think that the Indian facial overlay represents that ethnic group?					
9	In your opinion, how well do you think that the Jamaican facial overlay represents that ethnic group?					
10	Please rate how enhanced your medical training has become with the use of the ethnically diverse facial overlays.					
11	Please rate how comfortable you feel interacting with patients with varying ethnicities after using the facial overlays.					
12	Do you have any suggestions or changes that you would make to the facial overlays?					

Outcomes

Cost

The breakdown of all costs related to the manufacturing of the facial overlays is shown in Table 3. All cost estimates are in Canadian dollars (CAD), including local taxes.

**Table 3:** Cost breakdown to produce facial overlays. Total cost includes the one-time purchase products, and the materials used to make both facial overlays.

Cost Breakdown for Facial Overlays		
One-Time Purchase Products to Produce Facial Overlays		
Product	Cost (in CAD)	
Silc-Pig™ Silicone Pigments (sampler pack)	47.90	
Ease release 200	23.15	
TKBD-01 Mini Baby Doll Silicone Paint Trial Kit	59.83	
THI-VEX™	8.69	
Materials for East Indian Female Facial Overlay		
Material	Grams (g) Used	Cost (in CAD)
Ecotough™ PLA	580	16.38
Dragon Skin™ 10 NV silicone	660	30.00
Materials for Jamaican Male Facial Overlay		
Material	Grams (g) Used	Cost (in CAD)
Ecotough™ PLA	730	20.61
Dragon Skin™ 10 NV silicone	640	29.09
TOTAL COST	235.65	

User Feedback 

All 18 students filled out the survey, and the results were divided into quantitative data and qualitative data. The quantitative data are presented in Table 4. The results indicated difficulty in performing intubation, with an average rating of 2.29 on a scale of 1 to 5. Despite this functional flaw, the facial overlays were able to remain secure onto the simulation manikins with an average rating of 4.67. The eyes from the simulation manikin were still relatively visible, with an average rating of 3.67. However, the high standard deviation of 1.14 for this question should be noted. When asked how well the facial overlays represent their chosen ethnicities, the average response was between “represents well” (option 2) and “represents very well” (option 3), with the Jamaican facial overlay representing more accurately with an average response rating of 2.92 compared to the East Indian facial overlay with an average rating of 2.56. With regards to the attitudes toward the facial overlays enhancing students' medical education, the average response was 3.67 on a 5-point scale. Finally, when asked if the students felt more comfortable working with patients of different ethnicities after using the facial overlays, the average response was 4.67 on a 5-point scale.

**Table 4:** Qualitative data from facial overlay survey.

QUANTITATIVE DATA								
Question Number	Scale 1 (not effective) to 5 (very effective)					Total Responses	Average Response	Standard Deviation
	1	2	3	4	5			
1	4	7	4	1	1	17	2.29	1.1
	Scale 1 (poorly) to 5 (very well)							
	1	2	3	4	5			
3	0	3	6	3	6	18	3.67	1.14
4	0	1	0	3	14	18	4.67	0.77
	Scale 1 (not realistic) to 5 (very realistic)							
	1	2	3	4	5			
6	0	1	6	8	3	18	3.72	0.83
	Option 1*	Option 2**	Option 3***	Option 4****				
8	0	8	7	1		16	2.56	0.63
9	0	3	8	2		13	2.92	0.64
	Scale 1 (not enhanced) to 5 (enhanced)							
	1	2	3	4	5			
10	0	2	5	8	3	18	3.67	0.91
	Scale 1 (uncomfortable) to 5 (comfortable)							
	1	2	3	4	5			
11	0	0	2	2	14	18	4.67	0.69
* Does not represent at all								
** Somewhat represents								
*** Represents very well								
**** Not sure								

The qualitative data are presented in Table 5. From these results, it is evident that there was a lot of difficulty with performing intubations as the mouths of the facial overlays was noted as being too small and too stiff. It was also suggested to make the silicone thinner for ease of use, as well as add nasal holes so that oxygen can be administered for a more immersive training experience. Therefore, these design aspects will be refined in future iterations of the facial overlays. Additional noteworthy comments include the approval towards creating a more diverse medical training environment. 

**Table 5:** Quantitative data from facial overlay survey.

QUALITATIVE DATA										
Question Number	Comments									
2	- mouth too close together, hard to pull lips back (S1)					- mouth did not line up, silicone thick (S8)				
	- unable to open airway effectively (S2)					- manikin mouth is small already, the silicone on top makes it sticker to pass tube (S9)				
	- lips were easy to separate, however, it was difficult to insert an LMA (S3)					- mouth stiff (S11)				
	- only able to insert oral airway (S5)					- lips little too low, maybe thinner silicone (S12)				
	- oral cavity very rigid (S6)					- it was tough to use the LMA - mouth too small, tongue in way (S13)				
	- the mouth opening is narrow (S7)					- mouth opening has to be bigger width and open wider, it was harder to manipulate (S14)				
	- just the size of the lips was bigger, it was hard to put LMA (S15)					- the mask (LMA) was too large in comparison to the overlays mouth (S17)				
	- overlay was a little stiff to open mouth (S16)					- lips were stiff, mouth a bit narrow (S18)				
12	- more flexibility to open airway (S2)									
	- cut closer to the shape of eyes, include facial hair, portrays real life scenarios, nice to see different overlays being utilized in practice! (S3)									
	- nasal holes for nasal prongs (S4)									
	- please make an opening on the nose so O2 can be administered (S7)									
	- get the companies to make manikins more diverse to begin with - with your expertise (S9)									
	- maybe the overlay could be thinner, not so bulky (S11)									
	- consider thinner silicone to be more flexible for intubation (S12)									
	- bigger mouth, overlay adhesive so they stay on better (S13)									
	- would love manikins in other ethnicities (S15)									
	- excellent idea and clearly important (S18)									

## Discussion

In this technical report, the development process for cost-effective and diverse facial overlays that are to be used on top of current medical manikins was outlined. AM techniques and materials such as 3D scanning, 3D printing, and silicone were used to produce these facial overlays, keeping the manufacturing cost low. The cost to produce both facial overlays is CAD 235.65, which is significantly lower compared to the USD 65,000 - USD 85,000 needed to purchase high-fidelity medical manikins such as the SimMan® [4]. The facial overlays better represent the patient population in the Durham Region and aim to improve the quality of SBME as current medical manikins do not display much diversity. 

As there is not enough racial, ethnic, sex, and age diversity present in the current health care education curriculum, the development of diverse educational tools will greatly enhance the quality of medical training [12,13]. Different patient anatomies (sex and age), races, and ethnicities result in a variety of conditions, treatments, and diagnoses that healthcare learners must be exposed to, in order to develop and enhance their clinical skills [3]. Improving diversity in healthcare education has a positive effect on healthcare outcomes and patient experience [2,3]. Therefore, exposing healthcare learners to diverse settings during their training is an important aspect of healthcare education [2,3]. 

In addition to the diversity that the facial overlays add to current medical manikins, they are also practical as they do not completely impede the functionality of the manikins. It was critical that the eye areas were cut out of the facial overlays to allow for the learners to observe the realistic eyes on the medical manikin. The medical manikins have features that enable the eyes to blink and the pupils to dilate or constrict. This is pivotal to assessing a patient in a clinical setting, and thus, the eye area of the overlay should be unobstructed. Additionally, the facial overlays must be able to withstand intubation and therefore, they need to have their mouths open. However, it was found in the survey data that this function will need to be improved and can be achieved by making the mouths larger, more open, and not as stiff. 

In summary of the design process, the facial overlays were constructed using silicone and 3D printed molds based on the 3D scans of individuals from the chosen ethnic groups. The colouring added to the silicone was crafted to best match the skin colour of the volunteers that were 3D scanned. Silicone pigments were used to add details such as wrinkles, eyebrows, and lip colouring to the silicone base of the overlays. For stability features, Velcro was added to secure the facial overlay onto the medical manikins. Finally, for continuity, a neck overlay was added to cover the neck of the medical manikin so that the overlay would look realistic and better resemble a patient.

The next steps of this project are to add more accessories to the medical manikins to enhance the realism of the facial overlays. This includes wigs, jewellery, tattoos, and additional overlays for the arms, hands, and torso so that the entire manikin can reflect the ethnicity depicted by the facial overlay. Additionally, more facial overlays will be created to reflect additional minorities in the Durham Region. Finally, the efficacy of the facial overlays must be tested with more healthcare learners to assess whether the facial overlays improved or hindered their learning experience with the medical manikins.

## Conclusions

This technical report addresses the gap in SBME in which racial, ethnic, sex, and age diversity were not being represented with the current medical training manikins. Therefore, the process of developing facial overlays was described to fill this gap in SBME. The use of additive manufacturing techniques (3D scanning, 3D design, 3D printing, and silicone work) allowed for this to be completed cost-effectively. While the facial overlays were generally accepted as a means to improve diversity, some functional issues needed to be resolved before a formal and larger-scale evaluation of efficacy can be conducted.
